# Senescence‐associated DNA methylation is stochastically acquired in subpopulations of mesenchymal stem cells

**DOI:** 10.1111/acel.12544

**Published:** 2016-10-26

**Authors:** Julia Franzen, Anne Zirkel, Jonathon Blake, Björn Rath, Vladimir Benes, Argyris Papantonis, Wolfgang Wagner

**Affiliations:** ^1^ Helmholtz‐Institute for Biomedical EngineeringRWTH Aachen University Medical School52074AachenGermany; ^2^ Center for Molecular Medicine (CMMC)University of Cologne50931CologneGermany; ^3^ Genomics Core FacilityEuropean Molecular Biology Laboratory (EMBL)69117HeidelbergGermany; ^4^ Department for OrthopedicsRWTH Aachen University Medical School52074AachenGermany

**Keywords:** bisulfite sequencing, DNA methylation, epigenetics, human umbilical vein endothelial cell, mesenchymal stem cell, senescence

## Abstract

Replicative senescence has a major impact on function and integrity of cell preparations. This process is reflected by continuous DNA methylation (DNAm) changes at specific CpG dinucleotides in the course of *in vitro* culture, and such modifications can be used to estimate the state of cellular senescence for quality control of cell preparations. Still, it is unclear how senescence‐associated DNAm changes are regulated and whether they occur simultaneously across a cell population. In this study, we analyzed global DNAm profiles of human mesenchymal stem cells (MSCs) and human umbilical vein endothelial cells (HUVECs) to demonstrate that senescence‐associated DNAm changes are overall similar in these different cell types. Subsequently, an Epigenetic‐Senescence‐Signature, based on six CpGs, was either analyzed by pyrosequencing or by bar‐coded bisulfite amplicon sequencing. There was a good correlation between predicted and real passage numbers in bulk populations of MSCs (*R* ^2^ = 0.67) and HUVECs (*R* ^2^ = 0.97). However, when we analyzed the Epigenetic‐Senescence‐Signature in subclones of MSCs, the predictions revealed high variation and they were not related to the adipogenic or osteogenic differentiation potential of the subclones. Notably, in clonally derived subpopulations, the DNAm levels of neighboring CpGs differed extensively, indicating that these genomic regions are not synchronously modified during senescence. Taken together, senescence‐associated DNAm changes occur in a highly reproducible manner, but they are not synchronously co‐regulated. They rather appear to be acquired stochastically—potentially evoked by other epigenetic modifications.

## Introduction

Culture expansion of primary cells is limited to a certain number of cell divisions before entering replicative senescence (Hayflick, 1965). This is accompanied by proliferation arrest, increased cell size with ‘fried egg’ morphology, and alterations in gene expression and secretory profiles (Kuilman *et al*., 2010). Therefore, replicative senescence needs to be considered for quality control—particularly for therapeutic cell preparations, but also for basic research—to ensure reproducibility of results.

Mesenchymal stem cells (MSCs) are currently the most frequently used cell type in clinical trials (Trounson & McDonald, 2015). Due to their regenerative and immunomodulatory potential, they are applied for a huge variety of therapeutic approaches, for example, for orthopedic injuries, autoimmune diseases, or cardiovascular disorders (Kim & Cho, 2013). However, precise molecular markers for MSCs are still elusive (de Almeida *et al*., 2016) and there is a growing perception that these cell preparations are highly heterogeneous and only a small subfraction reflects multilineage differentiation potential (Phinney, 2012; Schellenberg *et al*., 2012). It has been demonstrated that the number of colony‐forming units and the number of cells with high adipogenic and osteogenic differentiation potential decline rapidly during culture expansion (Schellenberg *et al*., 2012, 2013). It is however unclear whether such functional differences are directly linked to heterogeneity in cellular aging—because it is not trivial to quantify the state of replicative senescence.

Senescence‐associated beta‐galactosidase (SA beta‐Gal) is frequently used to demarcate senescent cells (Dimri *et al*., 1995). However, SA beta‐Gal does not reflect the cause of senescence and this assay is not specific and difficult to quantify (Althubiti *et al*., 2014). The same applies to the analysis of upregulation of senescence‐associated genes, such as p16 and p21 (Herbig *et al*., 2004). Telomere attrition, which is generally perceived as an important mechanism that destabilizes chromatin integrity at later passages, can also be measured to estimate the proliferative capacity until senescence (Herbig *et al*., 2004; Hanzelmann *et al*., 2015). This approach is however hampered by the high inter‐ and intra‐organismal heterogeneity of telomere lengths (Bernadotte *et al*., 2016).

Cellular aging can also be tracked by epigenetic modifications. Replicative senescence is associated with highly reproducible changes in DNA methylation (DNAm): At specific CpG dinucleotides, the DNAm levels either increase or decrease over subsequent passages (Bork *et al*., 2010; Cruickshanks *et al*., 2013; Koch *et al*., 2013). We have recently described an Epigenetic‐Senescence‐Signature, which is based on DNA methylation levels at six specific CpG sites (Koch *et al*., 2012; Schellenberg *et al*., 2014). This signature enables prediction of the passage numbers or cumulative population doublings for MSCs or fibroblasts. It is however unclear whether these senescence‐associated modifications are equivalent in different cell types, how they are regulated, and whether they are functionally relevant *per se*.

In this study, we have analyzed MSCs and human umbilical vein endothelial cells (HUVECs) at different passages. Overall, senescence‐associated DNAm changes were similar in these two cell types. Subsequently, we subcloned MSCs to demonstrate that the epigenetic state of cellular senescence is very heterogeneous within cell preparations—and this was not associated with differences in the *in vitro* differentiation potential of the subclones. Last but not least, using a bar‐coded amplicon sequencing approach (Masser *et al*., 2013; Bernstein *et al*., 2015), we demonstrate that in clonally derived cell populations, neighboring CpG sites on the same DNA strand reveal stark fluctuation in DNAm levels, indicating that these modifications are not coherently modified, but rather stochastically acquired at specific sites in the genome.

## Results

### Comparison of epigenetic senescence in MSCs and HUVECs

**Figure 1:**
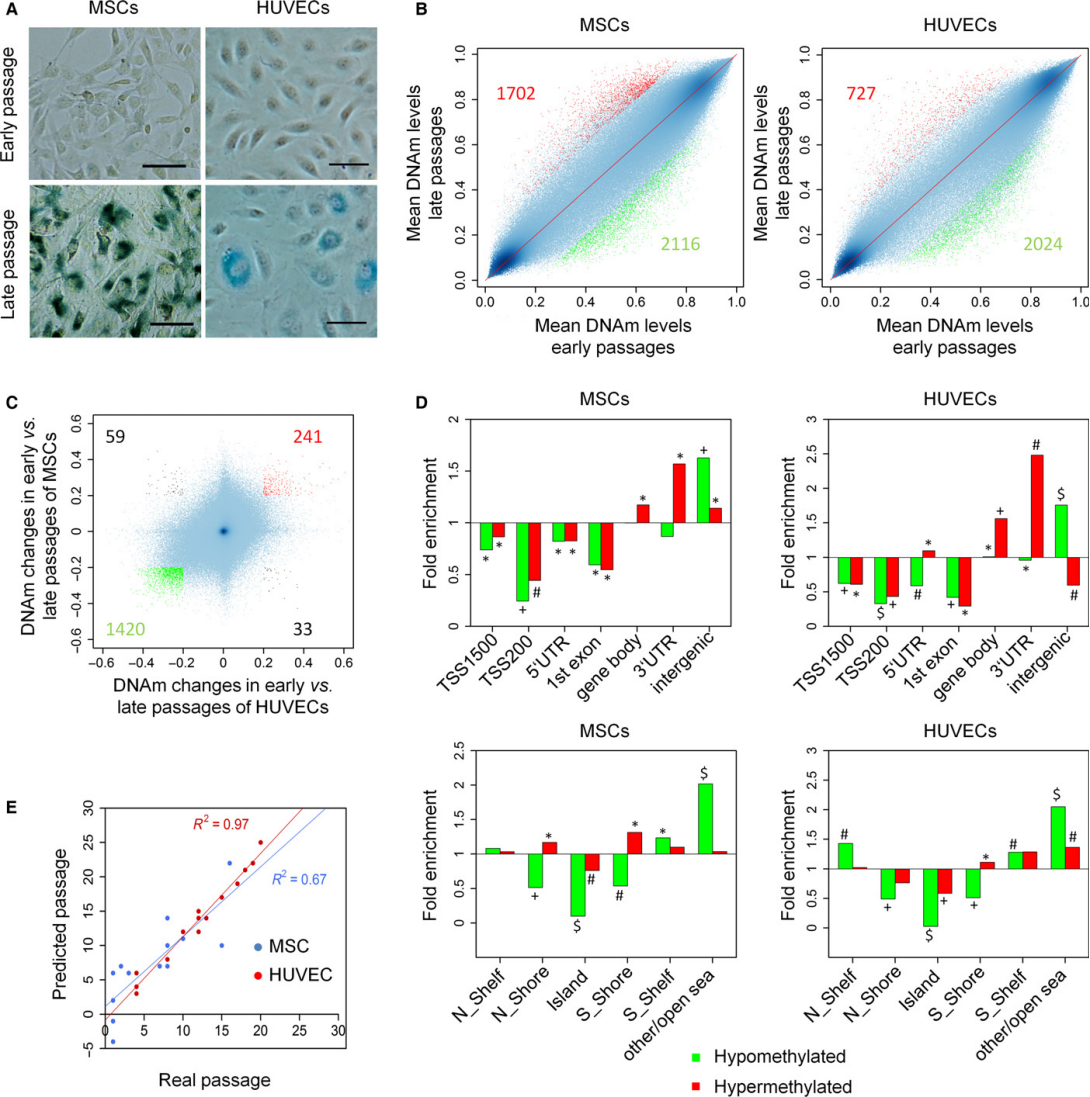
Comparison of senescence‐associated DNAm in different cell types. (A) Exemplary presentation of morphology and senescence‐associated beta‐galactosidase staining in MSCs and HUVECs at early and late passages (size bar = 100 μm). (B) The scatterplots compare mean DNAm levels (all CpGs on 450k Bead Chips) in either MSCs or HUVECs at early and late passages. CpGs with senescence‐associated hypo (green)‐ and hypermethylation (red) are indicated (20% change in DNAm level and adjusted *P*‐value < 0.05). (C) Direct comparison of senescence‐associated DNAm changes in MSCs and HUVECs (*R* ^2^ = 0.09). (D) Enrichment of senescence‐associated hypo (green)‐ and hypermethylated (red) CpGs in relation to associated gene regions or CpG islands (CGIs). A very similar pattern of enrichment was observed in both cell types at intergenic regions and apart from CGIs, whereas promoter regions and CGIs were highly significantly underrepresented (*P*‐values: *P < 0.01, #*P* < 10^−10^, +*P* < 10^−20^, $*P* < 10^−100^). (E) DNAm was analyzed by pyrosequencing at six senescence‐associated CpGs of the Epigenetic‐Senescence‐Signature in MSCs (13 samples of six donors) and HUVECs (14 samples of three donors) to estimate the state of cellular aging. Overall, predicted passage numbers correlated well with real passage numbers.

Mesenchymal stem cells (four donors) and HUVECs (three donors) were compared at early passages (P2 and P4, respectively) and near‐senescent passages (P7‐16 and P13‐20, respectively; Figs 1A and S1). DNAm profiles of these cell preparations were analyzed by 450k Illumina BeadChip microarrays: The dataset for HUVECs is deposited at gene expression omnibus (GSE82234)^1^, and for MSCs, we used our previously described dataset (GSE37066) (Koch *et al*., 2012). In both cell types, a similar number of CpGs revealed at least 20% changes in mean DNAm level during culture expansion (with an adjusted *P*‐value of < 0.05): Hypermethylation was observed at 1702 and 727 CpGs in MSCs and HUVECs, respectively; hypomethylation occurred at 2116 and 2024 CpGs, respectively (Fig. 1B). The majority of senescence‐associated DNAm changes were similar between MSCs and HUVECs. Particularly, CpGs with more than 20% hypomethylation showed a high overlap (1420 sites; Fig. 1C).

Senescence‐associated DNAm changes were enriched in three prime untranslated regions (3′UTR) and intergenic regions, whereas they were rather infrequent at CpGs associated with promoter regions (1500 or 200 bases upstream of transcription start sites [TSS1500, TSS200], 5′UTRs, or first exons; Fig. 1D). Furthermore, hypomethylation was hardly associated with CpG islands (CGIs) or neighboring shelf and shore regions (Fig. 1D). This pattern of enrichment was very similar in MSCs and HUVECs.

Subsequently, we tested our previously described Epigenetic‐Senescence‐Signature, which is based on DNAm levels at six senescence‐associated CpGs (Koch *et al*., 2012; Koch & Wagner, 2013). These CpGs are associated with the genes glutamate metabotropic receptor 7 (*GRM7*), calcium sensing receptor (*CASR*), selectin P (*SELP*), caspase 14 (*CASP14*), keratin‐associated protein 13‐3 (*KRTAP13‐3*), and PRAME family member 2 (*PRAMEF2*). The method was originally developed and trained on beta‐values of Illumina BeadChip data (Koch *et al*., 2012; Schellenberg *et al*., 2014), and the predicted passage numbers were overall rather overestimated by this model. To further improve the accuracy, we retrained the underlying linear models on a pyrosequencing dataset of previously published studies (Koch *et al*., 2012; Schellenberg *et al*., 2014) (Figs S2 and S3, Table S1). The predictions for passage numbers correlated with real passage numbers in our independent validation set (Table S2) for MSCs (13 samples of six donors; *R* ^2^ = 0.67) and HUVECs (14 samples of three donors; *R* ^2^ = 0.97; Fig. 1E). These results demonstrate that MSCs and HUVECs, which represent very different cell lineages, reflect overall similar and highly reproducible senescence‐associated DNAm changes.

### Bar‐coded bisulfite amplicon sequencing of the epigenetic‐senescence‐signature

**Figure 2:**
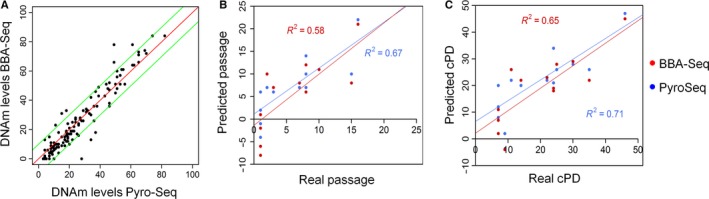
Bar‐coded bisulfite amplicon sequencing of the Epigenetic‐Senescence‐Signature. (A) Comparison of DNAm levels as determined either by pyrosequencing (PyroSeq) or bar‐coded bisulfite amplicon sequencing (BBA‐Seq) in all CpGs covered by the assays (bulk DNA of six different MSC preparations at early and late passages). These measurements were subsequently used to predict passage numbers (B) and cumulative population doublings (cPDs; (C), and the results of the Epigenetic‐Senescence‐Signature were similar based on PyroSeq (blue) or BBA‐Seq data (red).

The Epigenetic‐Senescence‐Signature was subsequently adapted for bar‐coded bisulfite amplicon sequencing (BBA‐Seq) (Masser *et al*., 2013; Bernstein *et al*., 2015). This method is more cost‐effective and less labor‐intensive for the multiplexed analysis of the six CpGs in multiple cell preparations. Furthermore, this sequencing approach provides insight into the variation of DNAm of neighboring CpG sites on the same DNA strands, whereas pyrosequencing provides only average DNAm levels for each CpG site. Nonetheless, DNAm measurements by pyrosequencing and bar‐coded bisulfite amplicon sequencing correlated overall very well (Fig. 2A).

These measurements were subsequently implemented in our Epigenetic‐Senescence‐Signature to estimate the state of cellular aging in six MSC preparations at early and late passage. Overall, the predicted and real values for passage numbers (Fig. 2B) and cumulative population doublings (cPDs; Fig. 2C) correlated for each of the methods. The slightly better performance of pyrosequencing data may be due to the fact that the models were originally trained on pyrosequencing data.

### Analysis of the epigenetic‐senescence‐signature in individual subclones

**Figure 3:**
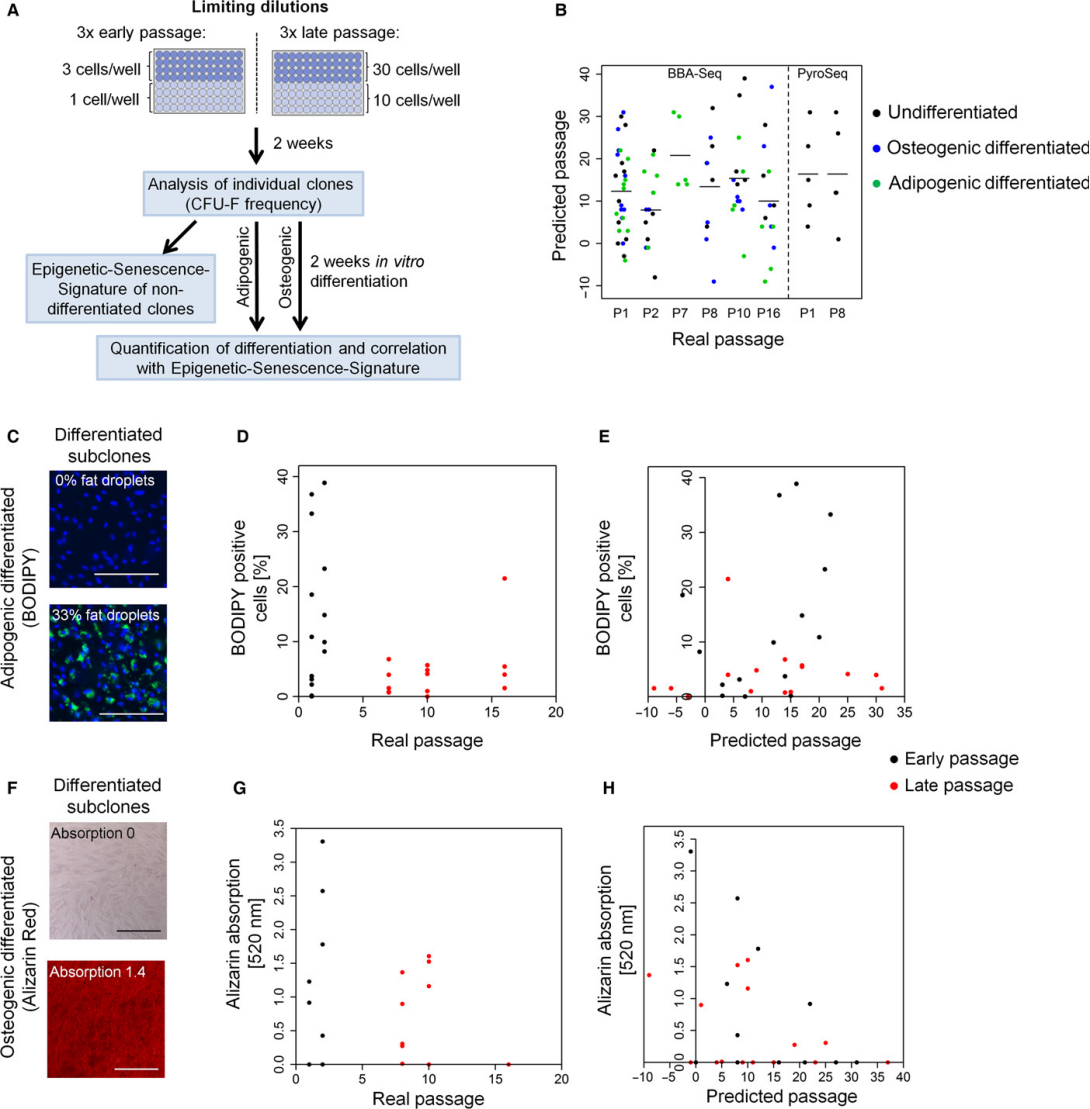
The Epigenetic‐Senescence‐Signature is not indicative in subclones of MSCs. (A) Schematic presentation of experimental design. (B) In total, 90 individual MSC colonies, which were statistically single‐cell‐derived, were analyzed by the Epigenetic‐Senescence‐Signature (BBA‐Seq: 30 CFU‐f undifferentiated, 30 colonies upon adipogenic differentiation, and 30 colonies upon osteogenic differentiation; for 10 colonies, the DNAm levels were validated by pyrosequencing). The predictions of the Epigenetic‐Senescence‐Signature were very heterogeneous between subclones and did not correlate with real passage numbers. (C) Individual MSC clones in 96‐well plates were differentiated toward adipogenic lineage for 2 weeks. The percentage of cells with fat droplets was quantified (stained with BODIPY, green; nuclei were counterstained with DAPI). Exemplary images are provided for a clone with low and high adipogenic differentiation potential (size bar: 200 μm). (D) MSC clones with higher adipogenic differentiation potential were particularly observed at early passages. (E) Predictions of passage numbers by the Epigenetic‐Senescence‐Signature did not correlate with adipogenic differentiation potential. (F) In analogy, other MSC clones were differentiated toward osteogenic lineage, and calcium phosphate deposition was quantified by Alizarin Red staining (size bar: 200 μm). (G) Clones with very high Alizarin Red staining were particularly observed at early passages. (H) No correlation was observed between predicted passage numbers and osteogenic differentiation potential.

To estimate heterogeneity in the epigenetic state of cellular aging within cell preparations, we subcloned MSCs at early (P1 – P2; *n* = 3 donors) and near‐senescent passages (P8, P10, P16; *n* = 3 donors) by limiting dilutions in 96‐well plates. After 2 weeks, fibroblastoid colony‐forming units (CFU‐f) were scored. The CFU‐f frequency declined during culture expansion as previously described (Schellenberg *et al*., 2012) (Fig. S4). For further analysis of individual clones, we focused only on those dilutions in which subclones were statistically single‐cell‐derived (Schellenberg *et al*., 2012) (Fig. 3A). The Epigenetic‐Senescence‐Signature was analyzed in 30 individual undifferentiated subclones (five clones per cell preparation) by BBA‐Seq. Results were validated for 10 selected colonies by pyrosequencing. In contrast to the bulk cultures, the predictions of passage numbers did not correlate at all with real passage numbers in single‐cell‐derived clones: The predictions varied between passage −10 and 40 in individual subsets (Fig. 3B). This may reflect the general perception that cellular aging is heterogeneous within MSCs. On the other hand, clonally derived subpopulations will only capture the epigenetic makeup of the initiating cell.

### Differentiation potential of subclones does not correlate with the epigenetic‐senescence‐signature

It has been demonstrated that the *in vitro* differentiation of MSCs toward adipogenic and osteogenic lineage declines continuously during culture expansion (Bonab *et al*., 2006; Noer *et al*., 2007; Schellenberg *et al*., 2012). Therefore, we hypothesized that clones, which are epigenetically predicted to be of higher passage, might also reveal lower *in vitro* differentiation potential. We performed additional limiting dilutions of the aforementioned MSC preparations, which were further differentiated toward adipogenic or osteogenic lineages for 2 weeks. The individual subclones revealed very heterogeneous *in vitro* differentiation potential as described in our previous work (Schellenberg *et al*., 2012; de Almeida *et al*., 2016) (Fig. S5). Adipogenic differentiation was estimated by the percentage of cells that acquired fat droplets during 2 weeks of differentiation (Fig. 3C). Subclones that gave a high fraction of cells with fat droplets (i.e. between 10% and 40%) were exclusively found at early passages (Fig. 3D). We then isolated DNA from 30 subclones with either higher or lower adipogenic differentiation potential and analyzed the Epigenetic‐Senescence‐Signature by BBA‐Seq (Fig. 3E). There was no clear association between adipogenic differentiation potential and epigenetic senescence predictions in individual subclones. Osteogenic differentiation was estimated by Alizarin Red staining of calcium phosphate precipitates (Fig. 3F). The measurements indicated that several subclones have higher propensity for osteogenic differentiation than others—at early rather than at later passages (Fig. 3G). We analyzed the Epigenetic‐Senescence‐Signature by BBA‐Seq in 30 additional subclones and could not observe any correlation between the osteogenic differentiation potential and predicted passages (Fig. 3H). Taken together, the *in vitro* differentiation potential is very heterogeneous between MSC subclones, but it is not reflected in the Epigenetic‐Senescence‐Signature.

### Methylation differences of neighboring CpG sites

**Figure 4:**
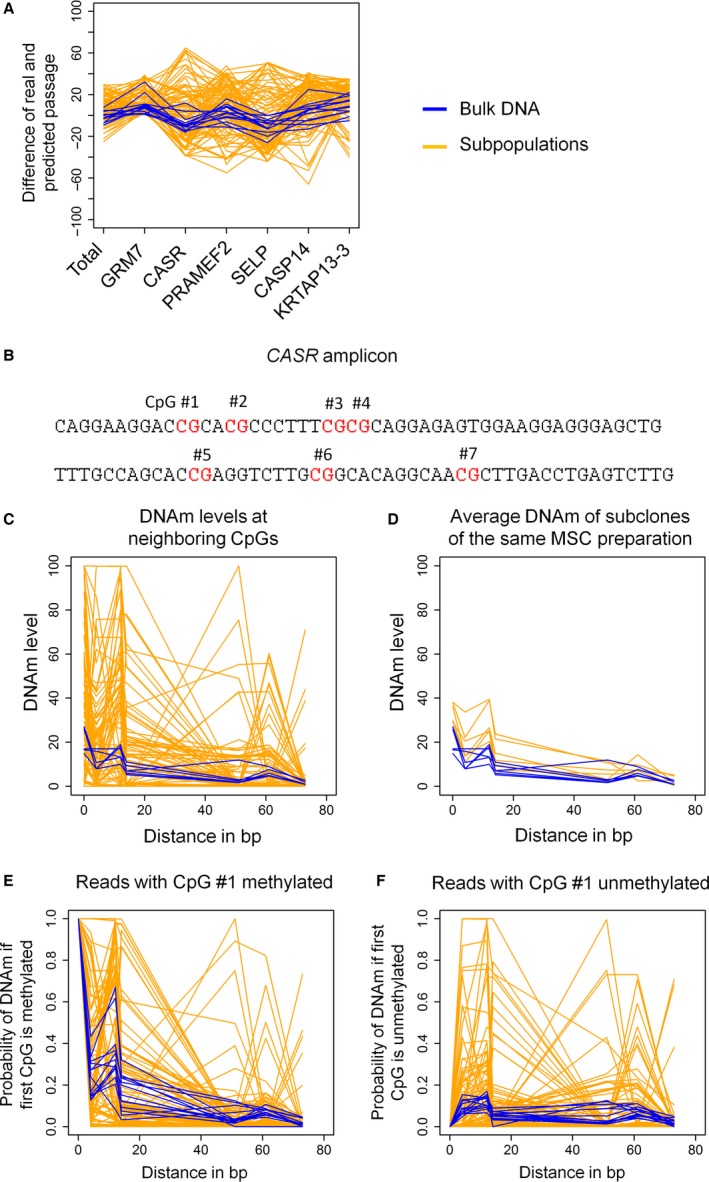
DNA methylation levels of neighboring CpGs are not co‐regulated. (A) The difference of predicted passage and real passage numbers was estimated either by combination of the six CpGs of the Epigenetic‐Senescence‐Signature (total) or by linear regression models for each of the individual CpGs (corresponding gene names are indicated). In bulk populations of MSCs (blue lines), predictions based on individual CpGs were overall consistent, whereas in DNA of the 90 individual subclones (yellow lines), the predictions based on individual CpGs varied extensively. (B) The amplicon of *CASR* comprised seven CpGs. (C) DNAm levels of these neighboring CpGs were similar in bulk MSCs (blue lines), whereas the DNAm levels varied a lot in subclones of MSCs (yellow lines). (D) Subsequently, we analyzed the mean DNAm levels at each CpG site for 15 subclones that corresponded to the same MSC preparation—and these values then closely resembled the DNAm levels of the bulk population. (E) The probability of DNAm was analyzed based on individual reads of BBA‐Seq where the first CpG was methylated. If the first CpG is methylated in bulk MSCs (blue), the CpGs in direct vicinity were more likely to be methylated, too. In contrast, analysis of individual subclones (yellow) reflected diverse methylation patterns, which were hardly related to DNAm levels at the first CpG. (F) In analogy, the same analysis was performed for those reads, where the first CpG was unmethylated.

To further analyze whether or not senescence‐associated CpGs are co‐regulated at different sites in the genome, we compared predictions based on linear regressions for each of the six individual CpGs of our Epigenetic‐Senescence‐Signature (Figs S2 and S3). In bulk MSCs, the predicted passage numbers for each CpG‐model overall corresponded to real passage numbers. Differences between predicted and real passages fluctuate closely around zero (Fig. 4A). In contrast, for individual subclones, there was a clear discrepancy in predictions based on the different senescence‐associated CpGs. DNAm levels at some CpGs were indicative for very early passage while other CpGs predicted the same subclone to be of much higher passage. Thus, senescence‐associated modifications are not co‐regulated at the six genomic locations.

Subsequently, we addressed the question whether neighboring CpGs are coherently modified. For example, the amplified region for the CpG site associated with the gene *CASR* comprised six additional CpG sites (Fig. 4B). In bulk populations of MSCs, the DNAm levels of neighboring sites were indeed similar. However, in the individual subclones, there were stark differences in the DNAm levels of neighboring sites, which may even range between 0% and 100% DNAm (Figs 4C and S6). This discrepancy of DNAm at neighboring CpG sites was observed in subpopulations of both early and late passages. Although these DNAm levels at neighboring CpGs seem to be randomly distributed, the mean DNAm levels across the different subclones closely reflected the DNAm patterns of the entire bulk populations (Fig. 4D).

In contrast to pyrosequencing analysis of DNAm levels, the BBA‐Seq methodology facilitates further analysis of neighboring CpG sites in the same read—corresponding to the same DNA strand. To this end, we have filtered for all reads of *CASR* in which the first CpG site—that was also relevant for the Epigenetic‐Senescence‐Signature—was methylated and then determined the percentage of reads in which the other neighboring CpGs were methylated, too. In bulk MSCs, the CpGs in close proximity to the first CpG, particularly CpG number 3, were methylated in about 20–60% of reads if CpG number 1 was methylated. However, already at CpG number 5, which is 51 bases downstream, no association with the DNAm at CpG number 1 was observed (Fig. 4E). Subsequently, we filtered for reads in which CpG number 1 was unmethylated (Fig. 4F): While the bulk samples suggested a homogeneously low probability of methylation if the first CpG is unmethylated, some subclones showed a high methylation frequency of neighboring sites. Overall, these findings indicate that the senescence‐associated changes are not concomitant at neighboring CpGs and that the epigenetic profile of the colony‐initiating cell remains prevalent in subclones even after colony formation.

## Discussion

It is yet unclear how senescence‐associated DNAm changes are regulated and whether they are at all functionally relevant. Several observations indicated that this process is tightly regulated: Senescence‐associated DNAm changes occur in a very reproducible manner, particularly in developmental genes (Bork *et al*., 2010; Cruickshanks *et al*., 2013; Koch *et al*., 2013; Hanzelmann *et al*., 2015), and they can be entirely reversed by reprogramming into induced pluripotent stem cells (iPSCs) (Koch *et al*., 2013; Frobel *et al*., 2014). On the other hand, it is conceivable that senescence‐associated DNAm changes are not directly mediated by a targeted molecular process, but that they rather reflect changes evoked by other epigenetic modifications or 3D chromatin structure.

MSCs and HUVECs represent different cell lineages with a different epigenetic makeup. In this regard, it is surprising that many CpG sites carry highly similar senescence‐associated DNAm changes and that the Epigenetic‐Senescence‐Signature provided extremely precise predictions of passage numbers in HUVECs (*R* ^2^ = 0.97) although it was not trained on data from this cell type. This might be due to the fact that for HUVECs multiple passages have been measured from the same donor (with less interindividual variation). Furthermore, MSCs might be more heterogeneous with regard to their subpopulations and therefore reveal higher intersample variation. In theory, it might be possible that senescence‐associated DNAm changes rather reflect changes in cellular composition—if different subpopulations have different proliferation rates, then this notoriously changes the heterogeneity during culture expansion (Wagner *et al*., 2010). On the other hand, the senescence‐associated DNAm changes are highly reproducible in different cell preparations, in different cell types, and apparently reversible by reprogramming into iPSCs. More importantly, our analysis of individual subclones demonstrated that these cannot be classified into subsets with ‘early passage type’ or ‘late passage type’ DNAm patterns—they revealed a clear discrepancy in senescence predictions based on the different senescence‐associated CpGs. These results clearly argue against the thesis that senescence‐associated DNAm patterns are only due to selection of particular cell lineages, but this aspect may still contribute to the observed changes in DNAm patterns.

Senescence‐associated DNAm changes were hardly observed in promoter regions or close to CGIs. Furthermore, it has previously been demonstrated that they are associated with repressive histone marks (Schellenberg *et al*., 2011; Fernandez *et al*., 2015) and with lamina‐associated domains (Cruickshanks *et al*., 2013; Hanzelmann *et al*., 2015). Thus, senescence‐associated DNAm changes seem to occur preferentially in heterochromatin, which may be less accessible to transcription factors or targeted modification. The enrichment of DNAm changes, particularly of senescence‐associated hypomethylation, apart from promoters and in more condensed chromatin, may also be the reason why these senescence‐associated DNAm changes are not generally reflected on gene expression level (Hanzelmann *et al*., 2015).

In this study, we demonstrate that the individual subclones of MSCs reflect extensive variation in senescence‐associated DNAm patterns. This can be attributed to the fact that each clone captures only the epigenetic makeup of the initial colony‐forming cell. In analogy, age‐related DNAm patterns do not reflect chronological age in cancer, which is a clonal disease (Lin & Wagner, 2015). Furthermore, predictions of our Epigenetic‐Senescence‐Signature were not related to adipogenic or osteogenic differentiation potential of the different subclones. These site‐specific DNAm changes are therefore not functionally relevant for the loss of *in vitro* differentiation potential at higher passages, which has been described by many authors before (Bonab *et al*., 2006; Noer *et al*., 2007; Schellenberg *et al*., 2012).

The DNAm levels in neighboring CpGs of MSCs usually reveal very similar changes upon replicative senescence, and we therefore anticipated that this is also observed in individual subclones. Recently, Lovkvist *et al*. (2016) suggested a model of collaborative methylation regulation where methylated regions attract methyltransferases, which methylate neighboring CpG sites, while nonmethylated regions attract demethylases that act more strongly on nearby CpGs. In this regard, it was unexpected that in MSC subclones very high differences exist in DNAm level of neighboring CpGs. Our measurements appear to be reliable, because the mean DNAm levels of different subclones closely resemble the pattern of the entire bulk population. These observations indicate that senescence‐associated DNAm at neighboring CpGs is not co‐regulated, but rather stochastically acquired. It is conceivable that chromatin loops, interactions with long noncoding RNAs (Kalwa *et al*., 2016), or chromatin conformation favor such modifications at specific sites in the genome. Chromatin reorganization is frequently observed in senescent cells (Zhang *et al*., 2003; Zhang & Adams, 2007; Chandra *et al*., 2015) and can be correlated to changes in different epigenetic compartments, like histone modifications and lamin‐associated domains (Shah *et al*., 2013). It may even be speculated, that similar mechanisms evoke age‐associated DNAm changes that are utilized for epigenetic aging clocks (Hannum *et al*., 2013; Horvath, 2013; Weidner *et al*., 2014)—albeit age‐associated and senescence‐associated DNAm changes are generally quite different (Bork *et al*., 2010; Lowe *et al*., 2016). Our findings of a stochastic DNA methylation mechanism of senescence‐associated CpG sites are in principle compatible with a theory by Holliday *et al*. that claimed stochastic commitment of cells to senescence already 40 years ago (Holliday *et al*., 1977): Parallel populations of MSCs may vary considerably in their longevity due to accumulating senescence‐associated epigenetic aberrations.

Taken together, our results demonstrate that senescence‐associated DNAm changes are acquired in a highly reproducible manner in different primary cell types such as MSCs and HUVECs. On the other hand, our results support the notion that they rather occur in heterochromatin, apart from promoter regions, and that they do not seem to be associated with *in vitro* differentiation potential. Last but not least, neighboring CpGs at a specific genomic region do not seem to be coherently methylated or demethylated, but the modifications rather occur in a stochastic manner at individual CpGs. It may therefore be anticipated that senescence‐associated DNAm changes are not mediated by a targeted molecular process and that they do not evoke the functional changes during replicative senescence. Either way, senescence‐associated DNAm pattern provides a very robust biomarker to estimate the state of cellular aging for quality control in primary cell preparations.

## Experimental procedures

### Cell culture procedures for MSCs

Mesenchymal stem cells were isolated from the bone marrow of patients after orthopedic surgery. All samples were taken after written consent from donors, and the study was approved by the ethics committee of RWTH Aachen University Medical School (permit number: EK300/13). Cells were flushed from bone marrow and cultured in Dulbecco's modified Eagle medium (DMEM, 1 g L^−1^ glucose; PAA, Pasching, Austria) supplemented with 1% penicillin/streptomycin (PAA), 1% l‐glutamine, 10% pooled human platelet lysate (Horn *et al*., 2010), and 0.1% heparin (5000 lU mL^−1^, Ratiopharm, Ulm, Germany) at 37 °C in an atmosphere with 5% CO_2_. Cells were harvested by trypsinization when reaching 80% confluency, counted, and re‐seeded in a density of 10 000 cells cm^−2^. Cumulative population doublings (cPDs) were calculated as described before (Cholewa *et al*., 2011). Quality control was performed for all MSC preparations with regard to immunophenotype (CD14^+^, CD29^+^, CD31^−^, CD34^+^, CD45^−^, CD73^+^, CD90^+^, and CD105^+^) and *in vitro* differentiation potential (osteogenic, adipogenic, and chondrogenic) (Pittenger *et al*., 1999; Koch *et al*., 2011).

### Culture procedures for HUVECs

Human umbilical vein endothelial cells of three different donors (Lonza, Basel, Switzerland) were cultured in Endopan 3 medium (PAN‐Biotech GmbH, Aidenbach, Germany) supplemented with 2% fetal bovine serum at 37 °C in an atmosphere with 5% CO_2_. Cells were harvested by trypsinization at ~80% confluency and re‐seeded at a density of 10 000 cells cm^−2^.

### Senescence‐associated β‐galactosidase staining

For staining of the senescent cells, the Abcam Senescence detection kit was used according to manufacturer's instructions (Abcam, Cambridge, UK).

### Limiting dilutions

Mesenchymal stem cells were seeded in limiting dilutions in 96‐well plates (1, 3, 10, and 30 cells per well, 48 replicas for each dilution). After 2 weeks, each well was scored and CFU‐f frequency was determined as described previously (Schellenberg *et al*., 2012). In brief, wells with at least 50% confluency were scored positive and CFU‐f frequency was estimated using the L‐Calc software (Stem Cell Technologies, Vancouver, Canada). Thereafter, some 96‐well plates were further differentiated toward adipogenic or osteogenic lineage for 2 weeks (Schellenberg *et al*., 2012; de Almeida *et al*., 2016). Upon adipogenic differentiation, fat droplets were stained with BODIPY (4,4‐difluoro‐1,2,5,7,8‐pentamethyl‐4‐bora‐3a,4a‐diaza‐s‐indacene), nuclei were counter stained with DAPI (4′,6‐diamidin‐2‐phenylindol; both Molecular Probes, Eugene, Oregon, USA), and the frequency of differentiated cells was estimated with ImageJ software (https://imagej.nih.gov/ij/) (Koch *et al*., 2011). Osteogenic differentiation was quantified by Alizarin Red staining and subsequent absorbance measurement at 520 nm with an Infinite 200 PRO Reader (Tecan Group Ltd., Männedorf, Switzerland) (Koch *et al*., 2011).

### DNA isolation and bisulfite conversion

Genomic DNA of MSCs was harvested with the NucleoSpin Tissue XS kit (Macherey‐Nagel, Düren, Germany). To isolate DNA of HUVECs, the cells were permeabilized (15 mm Tris/HCl ph7.6, 60 mm KCl, 15 mm NaCl, 4 mm CaCl_2_, 300 mm sucrose, 0.2% NP‐40, 0.5 mm beta‐mercaptoethanol), lysed (50 mm Tris/HCl ph8.0, 20 mm EDTA, 1% SDS) with addition of RNase A for 2 h at 37 °C and subsequently digested by proteinase K overnight at 37 °C. DNA of HUVECs was then purified by phenol/chloroform extraction. Bisulfite conversion was performed with the EZ DNA Methylation^™^ Kit (Zymo Research, Irvine (CA), USA).

### Infinium human methylation 450 bead chip analysis

Bisulfite converted DNA was analyzed on the Infinium HumanMethylation450 BeadChip according to the manufacturer's instructions (Illumina, San Diego, CA, USA). Hybridization and initial data analysis with the BeadStudio Methylation Module was performed at Life&Brain (Bonn, Germany). Raw data have been deposited at NCBIs Gene Expression Omnibus (GEO, http://www.ncbi.nlm.nih.gov/geo/, accession no.: GSE82234 and GSE37066). Significance of DNAm changes was estimated using the R limma package with an adjusted *P*‐value < 0.05. As additional cutoff, we used at least 20% difference in mean DNAm levels of early and late passages. Enrichment of differentially methylated CpG sites within USCS RefGene groups or enrichment in relation to USCS CpG islands was tested using hypergeometric distributions as described before (Koch *et al*., 2013).

### Analysis of the epigenetic‐senescence‐signature by pyrosequencing

DNA methylation levels of six CpG sites (associated with the genes *GRM7*,* CASP14*,* CASR*,* SELP*,* PRAMEF2,* and *KRTAP13‐3*) were analyzed by pyrosequencing as described in detail before (Koch *et al*., 2012; Koch & Wagner, 2013). In brief, bisulfite converted DNA was analyzed on a PyroMark Q96 ID System (Qiagen, Hilden, Germany) using sequencing primers described previously (Koch *et al*., 2012). Analyses of pyrosequencing results were performed with the PyroMark CpG SW 1.0 software (Qiagen). For HUVECs, analysis of the Epigenetic‐Senescence‐Signature was performed by Cygenia GmbH (Aachen, Germany). To further improve the precision of our signature, we have retrained the linear models on pyrosequencing results (previous models were generated based on beta‐values of Infinium HumanMethylation27 BeadChip data) as described in Figs S2 and S3. For each of the six relevant CpGs, linear regression models provide an estimate for passage number or cPD and these are subsequently averaged (Koch *et al*., 2012).

### Bar‐coded bisulfite amplicon sequencing of the epigenetic‐senescence‐signature

The sequences of each of the six CpG sites were amplified by PCR using primers containing overhangs for subsequent bar coding and library preparation (listed in Table S3). An adapted protocol of the PyroMark PCR Kit (Qiagen) was used for the PCR (50 cycles and 2 mm MgCl_2_). For each sample, amplicons of the six CpG sites were pooled and subsequently barcoded with 12‐bp bar codes using an adapted protocol of the NEXTflex^™^ 16S V1‐V3 Amplicon Seq Kit (Bioo Scientific, Austin, TX, USA). Pooled bar‐coded samples were analyzed on a MiSeq lane (Illumina) using 250PE mode for sequencing. Methylation levels of sequenced Illumina reads were calculated by the cytosine frequency at each CpG site divided by the total number of reads. The average number of reads per sample and genomic region was approximately 25 000 and only sequences that occurred at least 10 times were further considered.

## Funding

This work was particularly supported by the Else Kröner‐Fresenius‐Stiftung (AP and WW: 2010_A96; 2014_A193), by the German Research Foundation (WW: WA 1706/8‐1; AP: UoC Advancer Research grant of the DFG Excellence Initiative), by the German Ministry of Education and Research (WW: OBELICS, 01KU1402B), by the Interdisciplinary Center for Clinical Research (WW: IZKF; O‐1) within the faculty of Medicine at the RWTH Aachen University, and by the CMMC core funding (AP).

## Conflict of interest

Wolfgang Wagner is cofounder of Cygenia GmbH (www.cygenia.com), which can provide service for the Epigenetic‐Senescence‐Signature to other scientists. All other authors do not have a conflict of interest to declare.

## Author contributions

JF performed experiments on MSCs contributed to experimental design, data analysis, and manuscript preparation; AZ performed experiments on HUVECs and contributed to manuscript preparation. JB and VB contributed to experimental design, execution, and data analysis of Illumina sequencing. BR contributed important material. AP and WW contributed to experimental design, data analysis, and manuscript preparation.

## Supporting information


**Fig. S1** Quality control and long‐term growth curves of MSCs.
**Fig. S2** Linear regression models for prediction of passage numbers.
**Fig. S3** Linear regression models for prediction of cumulative population doublings.
**Fig. S4** CFU‐f frequencies of cell preparations used for limiting dilutions.
**Fig. S5** *In vitro* differentiation of subclones of the same MSC preparations.
**Fig. S6** DNA methylation levels of neighboring CpGs in *GRM7*.
**Table S1** Training dataset for an Epigenetic‐Senescence‐Signature.
**Table S2** Validation dataset for an Epigenetic‐Senescence‐Signature.
**Table S3** Primer for BBA‐Seq analysis.

## Note

1 The following link has been created to allow review of record GSE82234, while it remains in private status: http://www.ncbi.nlm.nih.gov/geo/query/acc.cgi?token=gpuvgecmftynfcj&acc=GSE82234
