# Supplementary material for: Senescence‐associated DNA methylation is stochastically acquired in subpopulations of mesenchymal stem cells
Source: Aging Cell. 2016 Oct 26;16(1):183–91. doi: 10.1111/acel.12544 (PMC5242294; doi:10.1111/acel.12544)
Supplement: Supplementary file 1 — Fig. S1 Quality control and long‐term growth curves of MSCs. Fig. S2 Linear regression models for prediction of passage numbers. Fig. S3 Linear regression models for prediction of cumulative population doublings. Fig. S4 CFU‐f frequencies of cell preparations used for limiting dilutions. Fig. S5 In vitro differentiation of subclones of the same MSC preparations. Fig. S6 DNA methylation levels of neighboring CpGs in GRM7. Table S1 Training dataset for an Epigenetic‐Senescence‐Signature. Table S2 Validation dataset for an Epigenetic‐Senescence‐Signature. Table S3 Primer for BBA‐Seq analysis. [file ACEL-16-183-s001.pdf]

Supplemental Material

Senescence-Associated DNA Methylation is Stochastically Acquired in Subpopulations of Mesenchymal Stem Cells

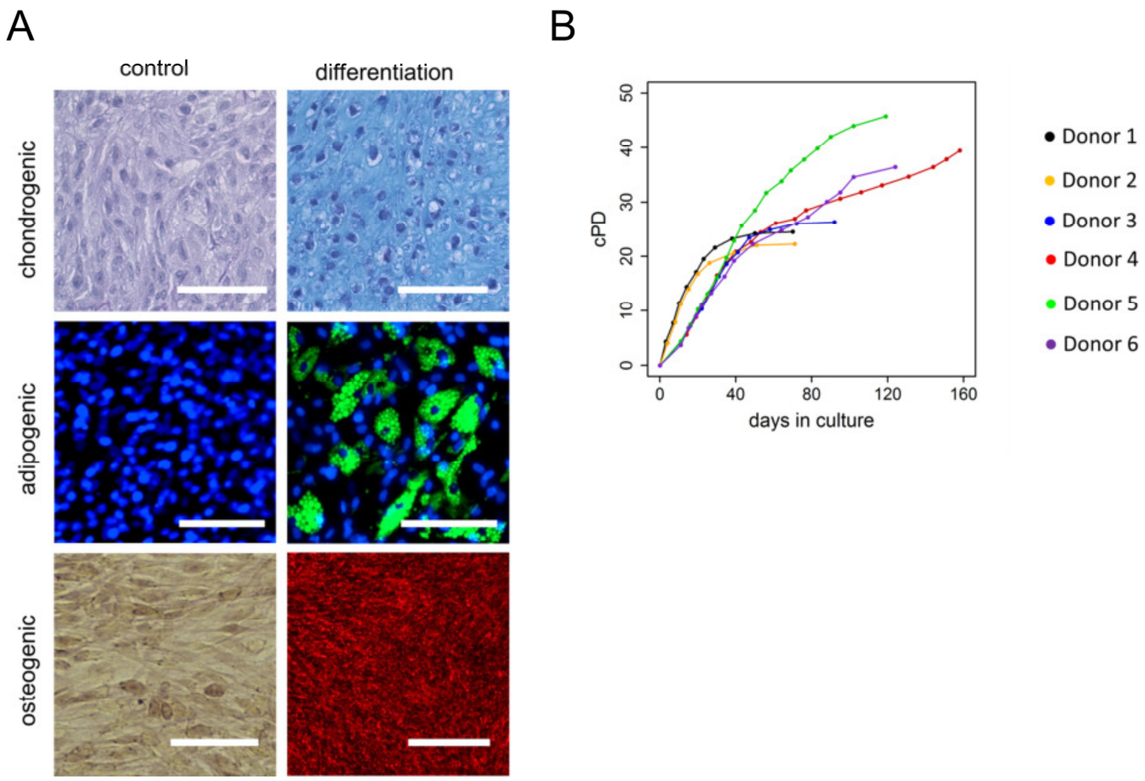

Supplemental Fig. 1: Quality control and long-term growth curves of MSCs.
A) The *in vitro* differentiation potential towards chondrogenic, adipogenic and osteogenic lineages was validated for all donors: chondrogenic differentiation was analyzed by staining of glycosaminoglycans with Alcian Blue and PAS; adipogenic differentiation was analyzed by staining of lipid droplets with BODIPY and nuclei counterstained with DAPI; and osteogenic differentiation estimated by staining of calcium phosphate precipitates by Alizarin Red staining (size bar: 100 µm). **B)** Cumulative population doublings (cPDs) are plotted against the days in culture to assess growth curves.

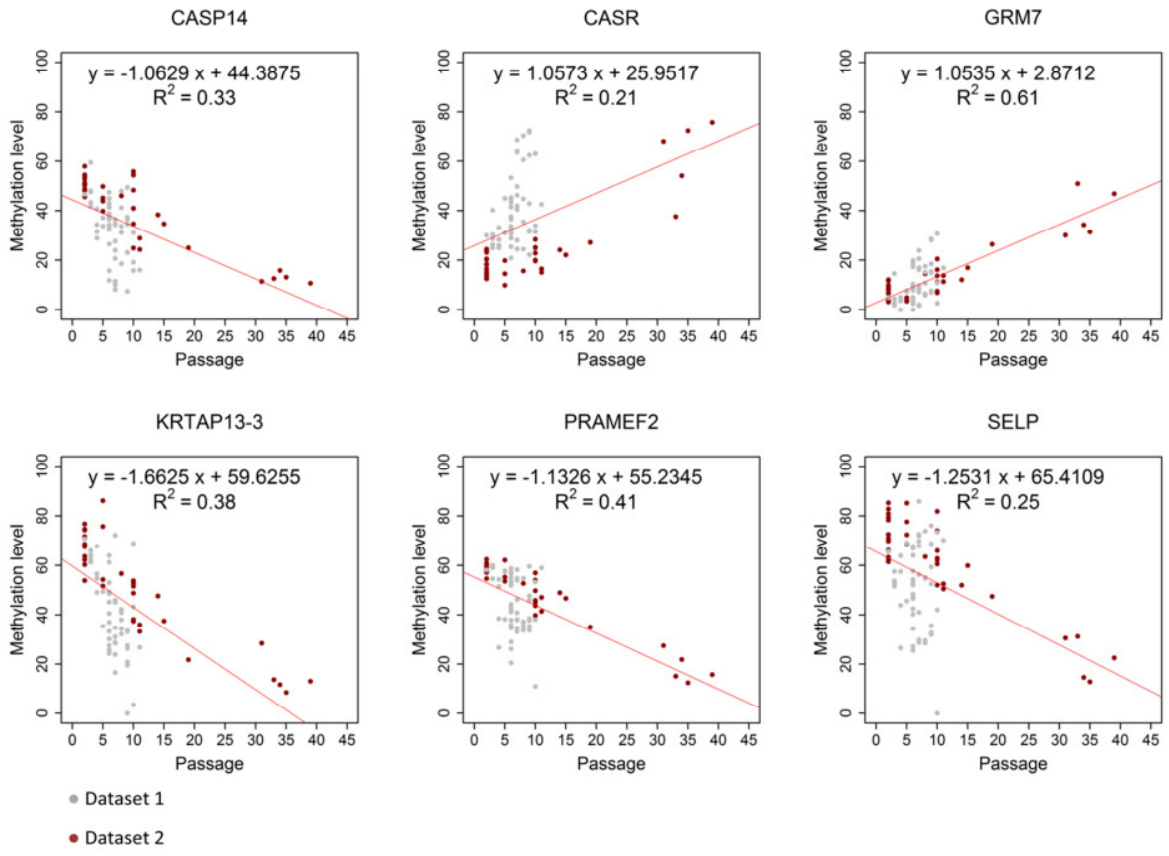

Supplemental Fig. 2: Linear regression models for prediction of passage numbers.
Linear regression models for the six CpG sites of the Epigenetic-Senescence-Signature were recalculated based on pyrosequencing data from two former studies: dataset 1 (Koch *et al*. 2012) and dataset 2 (Schellenberg *et al*. 2014). For each of the six CpGs (corresponding genes are indicated) the DNAm levels were plotted against the passage numbers to derive linear regressions models. Based on these equations predictions can be made for independent samples – and the Epigenetic-Senescence-Signature corresponds to the mean of the six predicted values.

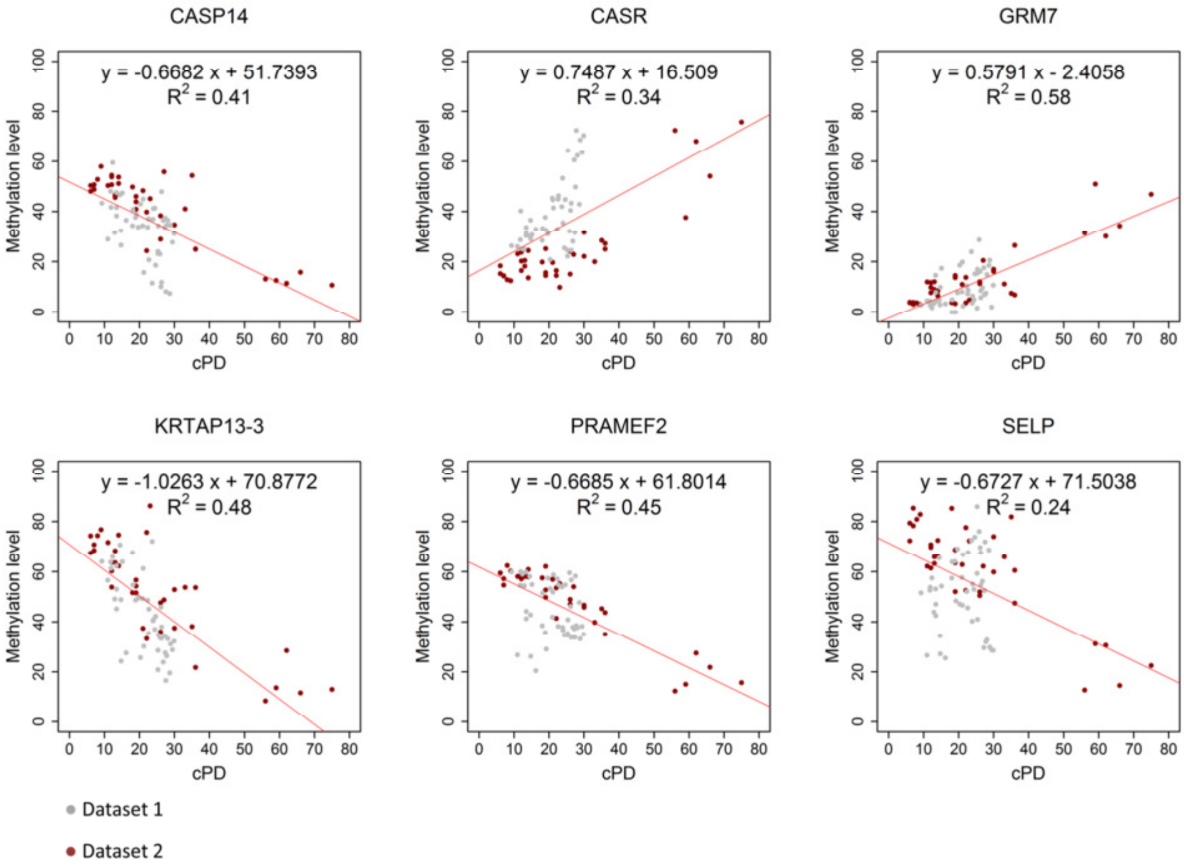

Supplemental Fig. 3: Linear regression models for prediction of cumulative population doublings. Linear regression models for the six CpG sites of the Epigenetic-Senescence-Signature were recalculated based on pyrosequencing data from two former studies: dataset 1 (Koch *et al.* 2012) and dataset 2 (Schellenberg *et al.* 2014). DNAm levels were plotted against cumulative population doublings (cPD) number for linear regressions in analogy to Supplemental Figure S2.

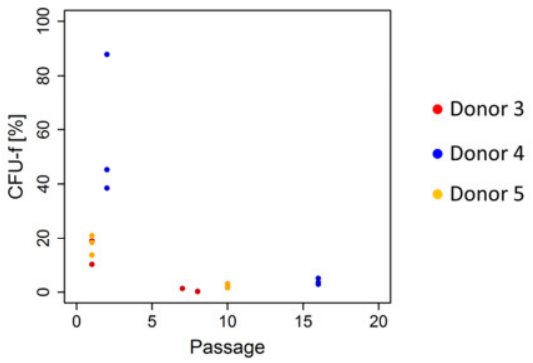

Supplemental Fig. 4: CFU-f frequencies of cell preparations used for limiting dilutions. The frequency of fibroblastoid colony forming units (CFU-f) within the MSC preparations was determined in limiting dilutions using Poisson statistics (Schellenberg *et al.* 2012). In all three MSC preparations the percentage of cells that are capable of colony formation decays significantly at later passages.

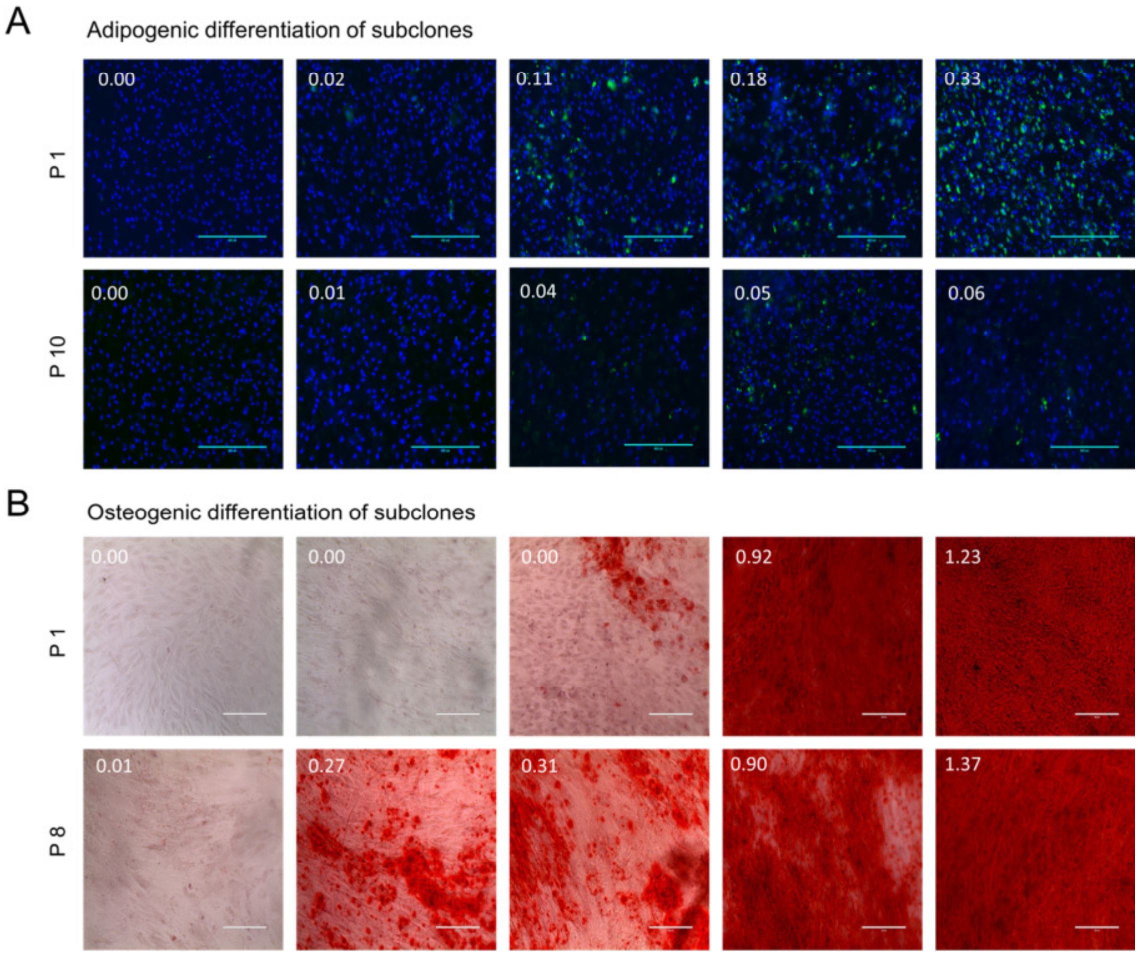

Supplemental Fig. 5: *In vitro* differentiation of subclones of the same MSC preparations.
This figure depicts different subclones upon either adipogenic differentiation **(A)** or osteogenic differentiation **(B)**, which were selected for subsequent analysis by the Epigenetic-Senescence-Signature. For adipogenic differentiation, the percentage of cells with fat droplets (stained by BODIPY, counterstained by DAPI) is indicated in each subpanel (all from donor 5; size bar: 400 µm). Osteogenic differentiation (all donor 3; size bar: 200 µm) was estimated by absorption of Alizarin Red staining (520 nm; measurements indicated).

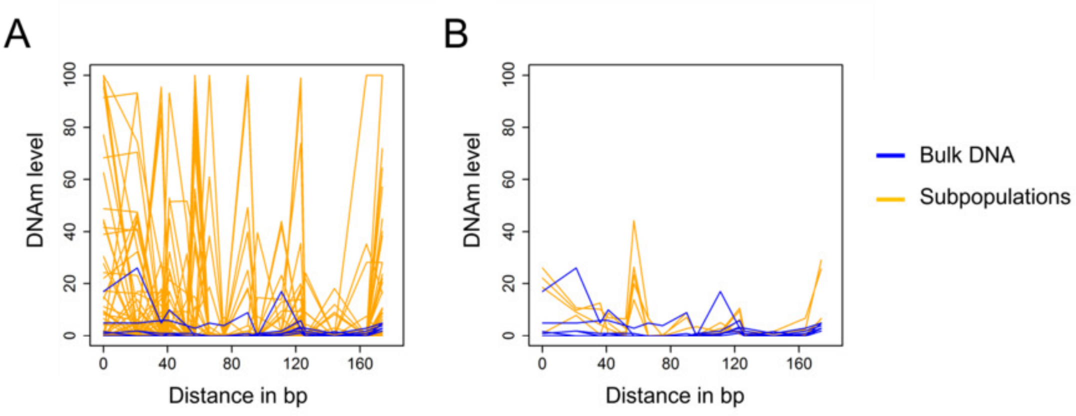

Supplemental Fig. 6: DNA-methylation levels of neighboring CpGs in *GRM7*.
The amplicon of *GRM7* comprises 22 neighboring CpGs on a total length of 174 bp. **A)** DNAm levels of individual CpGs are demonstrated for all 90 undifferentiated and differentiated MSC subpopulations (yellow) in comparison to DNAm levels of bulk DNA (blue). **B)** Mean DNAm levels at each CpG site of all subpopulations that correspond to the same MSC sample (yellow) are compared to methylation levels of bulk DNA (blue; in analogy to Figure 4).

Supplemental Tab. 1: Training dataset for an Epigenetic-Senescence-Signature.

Sample	cell type	Passage Nr	cPD	Dataset	*GRM7*	*CASR*	*PRAMEF2*	*SELP*	*CASP 14*	*KTRAP 13-3*
1	Fibroblast	2	7	1	3	14	55	85	49	71
2	Fibroblast	2	7	1	4	14	57	78	51	69
3	Fibroblast	2	6	1	4	15	59	79	50	74
4	Fibroblast	2	9	1	3	13	60	83	58	77
5	AT MSC	2	6	1	4	18	59	72	48	68
6	AT MSC	2	8	1	4	13	62	81	53	74
7	Fibroblast	39	75	1	47	76	16	23	11	13
8	Fibroblast	33	59	1	51	38	15	31	13	14
9	Fibroblast	34	66	1	34	54	22	15	16	12
10	Fibroblast	31	62	1	30	68	27	31	11	29
11	AT MSC	35	56	1	31	72	13	13	13	9
12	AT MSC	19	36	1	26	27	35	48	25	22
13	AT MSC	5	18	1	4	20	58	85	50	52
14	AT MSC	5	22	1	4	15	54	78	40	76
15	AT MSC	5	23	1	5	10	55	72	45	86
16	AT MSC	5	19	1	3	15	62	69	44	54
17	AT MSC	10	30	1	16	32	46	74	35	53
18	AT MSC	10	35	1	8	28	45	82	54	38
19	AT MSC	10	36	1	7	25	44	61	25	54
20	AT MSC	10	33	1	11	20	40	66	41	54
21	BM MSC	2	13	1	12	18	59	63	46	64
22	BM MSC	2	14	1	7	24	58	66	54	75
23	BM MSC	2	12	1	10	24	57	61	51	64
24	BM MSC	2	11	1	12	23	58	62	50	72
25	BM MSC	2	12	1	12	20	58	70	54	54
26	BM MSC	2	14	1	9	14	61	73	51	62
27	BM MSC	2	13	1	9	21	58	66	47	68
28	BM MSC	2	12	1	8	17	58	71	54	60
29	BM MSC	11	26	1	11	15	47	51	29	36
30	BM MSC	14	26	1	12	24	49	52	38	48
31	BM MSC	11	22	1	14	17	42	53	24	33
32	BM MSC	10	19	1	14	25	50	52	41	52
33	BM MSC	10	27	1	21	23	54	62	56	49
34	BM MSC	15	30	1	17	22	47	60	35	38
35	BM MSC	8	19	1	15	16	53	63	46	57
36	BM MSC	10	21	1	11	20	57	63	48	38
37	BM MSC	2	14	2	5	30	58	66	47	71
38	BM MSC	4	18	2	5	33	52	64	41	55
39	BM MSC	5	20	2	8	31	57	69	34	49
40	BM MSC	6	21	2	3	42	59	67	16	30
41	BM MSC	2	14	2	5	30	58	66	47	71
42	BM MSC	4	18	2	5	33	52	64	41	55
43	BM MSC	5	20	2	8	31	57	69	34	49
44	BM MSC	7	24	2	24	42	54	73	12	72
45	BM MSC	8	25	2	15	45	55	74	19	42
46	BM MSC	9	26	2	29	32	60	76	23	34
47	BM MSC	10	NA	2	3	34	11	0	19	69
48	BM MSC	2	14	2	5	30	58	66	47	71
49	BM MSC	6	26	2	9	43	57	68	12	33
50	BM MSC	2	14	2	5	30	58	66	47	71
51	BM MSC	7	26	2	16	41	46	70	10	46
52	BM MSC	11	NA	2	17	43	59	70	16	27
53	BM MSC	2	14	2	5	30	58	66	47	71
54	BM MSC	7	28	2	7	50	58	72	8	17
55	BM MSC	9	29	2	19	43	48	69	7	20
56	BM MSC	10	NA	2	11	32	53	73	16	43
57	BM MSC	5	23	2	7	45	38	46	37	36
58	BM MSC	6	24	2	4	53	38	40	37	41
59	BM MSC	7	25	2	17	64	41	41	34	35
60	BM MSC	9	28	2	8	72	39	32	35	26
61	BM MSC	10	NA	2	31	63	38	42	31	3
62	BM MSC	5	23	2	7	45	38	46	37	36
63	BM MSC	6	24	2	4	53	38	40	37	41
64	BM MSC	8	27	2	18	61	37	30	34	34
65	BM MSC	9	28	2	15	62	38	33	37	33
66	BM MSC	7	29	2	2	64	38	29	28	29
67	BM MSC	7	29	2	21	69	33	30	35	31
68	BM MSC	8	30	2	11	70	35	29	31	32
69	BM MSC	9	NA	2	28	72	46	36	49	0
70	BM MSC	4	18	2	0	28	39	57	34	68
71	BM MSC	7	23	2	9	46	55	68	21	45
72	BM MSC	8	24	2	7	36	37	55	41	41
73	BM MSC	9	25	2	15	22	35	59	23	21
74	BM MSC	4	18	2	0	28	39	57	34	68
75	BM MSC	6	24	2	5	25	35	53	41	37
76	BM MSC	7	27	2	10	28	38	56	37	24
77	BM MSC	7	25	2	2	25	35	86	24	29
78	BM MSC	8	26	2	6	24	34	55	48	38
79	BM MSC	9	27	2	7	32	34	52	38	28
80	BM MSC	3	12	2	5	29	55	53	48	64
81	BM MSC	4	13	2	4	25	54	44	32	49
82	BM MSC	6	21	2	8	45	42	58	34	49
83	BM MSC	3	9	2	3	26	60	52	43	64
84	BM MSC	4	11	2	5	26	27	27	29	57
85	BM MSC	6	19	2	0	33	29	48	39	
86	BM MSC	3	12	2	2	25	59	58	59	66
87	BM MSC	6	19	2	0	38	36	64	38	49
88	BM MSC	3	12	2	3	40	60	54	42	61
89	BM MSC	6	21	2	20	45	52	47	45	61
90	BM MSC	3	13	2	15	30	60	53	48	62
91	BM MSC	6	20	2	19	21	53	47	43	26
92	BM MSC	6	16	2	3	33	21	26	37	28
93	BM MSC	6	15	2	17	31	55	54	47	64
94	BM MSC	6	15	2	10	29	26	27	27	24
95	BM MSC	6	15	2	3	37	43	34	32	49
96	BM MSC	6	13	2	4	31	44	47	42	53
97	BM MSC	6	13	2	8	34	41	43	38	45
98	BM MSC	6	22	2	10	33	57	52	34	49

Dataset 1 and 2 refer to pyrosequencing studies from Koch *et al.* 2012 and Schellenberg *et al.* 2014, respectively. Methylation values of the six CpG sites are presented in percent. NA = not analyzed.

Supplemental Tab. 2: Validation dataset for an Epigenetic-Senescence-Signature.

Sample	cell type	Passage Nr	cPD	Sample type	*GRM7*	*CASR*	*PRAMEF2*	*SELP*	*CASP14*	*KTRAP13-3*
1	BM MSC	1	7	bulk DNA	4	21	56	62	38	50
2	BM MSC	8	25	bulk DNA	8	24	46	54	26	19
3	BM MSC	1	9	bulk DNA	4	17	65	74	53	51
4	BM MSC	8	24	bulk DNA	10	24	51	61	31	32
5	BM MSC	15	35	bulk DNA	28	29	50	70	29	27
6	BM MSC	1	7	bulk DNA	28	25	51	65	45	44
7	BM MSC	10	30	bulk DNA	7	27	33	55	31	24
8	BM MSC	1	7	bulk DNA	4	19	53	69	49	49
9	BM MSC	16	46	bulk DNA	25	28	22	37	13	11
10	BM MSC	3	14	bulk DNA	5	18	46	49	36	37
11	BM MSC	8	24	bulk DNA	38	23	36	53	41	18
12	BM MSC	2	11	bulk DNA	4	19	43	61	28	26
13	BM MSC	7	22	bulk DNA	10	23	46	65	34	22
14	HUVEC	4	NA	bulk DNA	1	21	47	44	54	43
15	HUVEC	10	NA	bulk DNA	1	23	34	44	27	23
16	HUVEC	12	NA	bulk DNA	2	28	35	35	26	18
17	HUVEC	15	NA	bulk DNA	12	32	29	39	26	13
18	HUVEC	19	NA	bulk DNA	9	39	23	35	19	2
19	HUVEC	20	NA	bulk DNA	5	53	18	24	19	5
20	HUVEC	4	NA	bulk DNA	1	13	36	32	50	44
21	HUVEC	12	NA	bulk DNA	10	20	25	30	35	16
22	HUVEC	17	NA	bulk DNA	7	21	18	38	20	3
23	HUVEC	18	NA	bulk DNA	10	27	18	34	17	7
24	HUVEC	4	NA	bulk DNA	5	14	52	39	48	42
25	HUVEC	8	NA	bulk DNA	7	23	48	40	39	30
26	HUVEC	12	NA	bulk DNA	5	26	35	26	40	30
27	HUVEC	13	NA	bulk DNA	18	26	33	32	41	26

Methylation values of the six CpG sites were generated by pyrosequencing and are presented in percent. NA = not analyzed.

Supplemental Tab. 3: Primer for BBA-Seq analysis.

Primer name	Primer sequence
GRM7-handle-for	CTCTTTCCCTACACGACGCTCTTCCGATCTTTGGGATTATTGTTGATTT
GRM7-handle-rev	CTGGAGTTCAGACGTGTGCTCTTCCGATCTCCCCTACTACCTACTAAAAATA
CASR-handle-for	CTCTTTCCCTACACGACGCTCTTCCGATCTTGTAATAGGTATTTGGTTGTAGT
CASR-handle-rev	CTGGAGTTCAGACGTGTGCTCTTCCGATCTCCCAAACTCTTACTCATTCTA
PRAMEF2-handle-for	CTCTTTCCCTACACGACGCTCTTCCGATCTTTTGAGGGTATTTAGAAGAGAT
PRAMEF2-handle-rev	CTGGAGTTCAGACGTGTGCTCTTCCGATCTTCCCTAACTAACTAACTACTAATC
SELP-handle-for	CTCTTTCCCTACACGACGCTCTTCCGATCTAGAAGGTAGAAAATTAGTAGAGTT
SELP-handle-rev	CTGGAGTTCAGACGTGTGCTCTTCCGATCTCAACATAAAACTCCATAACTA
CASP14-handle-for	CTCTTTCCCTACACGACGCTCTTCCGATCTTTGGAGATTTAGTGAGATAATA
CASP14-handle-rev	CTGGAGTTCAGACGTGTGCTCTTCCGATCTAACAAAACAAATAACCCATATA
KRTAP13-3-handle-for	CTCTTTCCCTACACGACGCTCTTCCGATCTGAGATTTGTTGGAGGTTTAA
KRTAP13-3-handle-rev	CTGGAGTTCAGACGTGTGCTCTTCCGATCTCCCAATAAAAAACAACTCC

Handle sequences for the barcoding PCR are depicted in red.
